# Centering Undocumented Immigrants: A Cross-Sectional Study of Sexual and Reproductive Health of Undocumented Asian and Latinx Immigrants in California

**DOI:** 10.1007/s10903-026-01919-7

**Published:** 2026-04-28

**Authors:** May Sudhinaraset, Angubeen Khan, Hye Young Choi, Irving Ling

**Affiliations:** 1 University of California, Los Angeles, Los Angeles, USA; 2 University of Michigan–Ann Arbor, Ann Arbor, USA; 3 Yale University, New Haven, USA

**Keywords:** Immigration, Sexual and reproductive healthcare, Undocumented, Health insurance

## Abstract

Undocumented immigrants face significant barriers to sexual and reproductive healthcare (SRH); however, there is a dearth of empirical data among this population. This study aims to examine the role of health insurance and SRH use among undocumented immigrants in California. This study uses cross-sectional survey data conducted between July 2020 and May 2022 in California. The final sample includes undocumented female and gender non-conforming adults aged 18–40 years (*N* = 123). The outcomes of interest include the use of a range of SRH services (i.e., pap smear, pelvic exams, STI testing, contraception, pregnancy tests, or abortion services), ever seeking advice related to SRH, place where SRH services like contraception were obtained, and how respondents paid for services. Bivariate and multivariate analyses were conducted to assess the associations between insurance status and SRH. Overall, 55% of participants used at least one SRH service in the past 12 months. In multivariable results, compared to those with private insurance, those who were uninsured were less likely to seek SRH advice (OR = 0.07; CI: 0.012–0.389) and receive any SRH use in the past 12 months (OR = 0.055; CI: 0.012–0.256). Those with public health insurance were also less likely to receive any SRH in the past 12 months and pay for contraception using insurance compared to those with private insurance. A college degree increased the likelihood of receiving any SRH in the past 12 months. Findings suggest that lack of insurance is strongly associated with lower SRH utilization.

## Background

Over 44 million foreign-born individuals live in the U.S [1]. and represent 17% of reproductive aged women and 23% of births [2]. Among reproductive-aged immigrant women aged 18–49 years, about 3.7 million are undocumented [3]. Immigrants are at risk of inequities in access to and use of health care services, including sexual and reproductive healthcare (SRH), which can result in differences in health status and outcomes over time [4]. Factors, such as exclusion from public health programs, restrictive immigration policies and immigration enforcement activities especially increase the vulnerability of undocumented immigrants that further undermines their access to and utilization of sexual and reproductive healthcare [5]. For example, immigration raids and restrictive immigration policies have been associated with increased risk of adverse birth outcomes [6, 7] and decreased prenatal care utilization among immigrant women [8]. The increasing landscape of restrictive reproductive policies in the US may disproportionately impact immigrants, including those who are undocumented [9].

The Affordable Care Act (ACA) explicitly excludes undocumented immigrants from participating in state exchange marketplace coverage. While the federal government has jurisdiction over the designation of legal status, states are increasingly responsible for implementing and developing policies that impact the everyday lives of immigrants, including providing health care coverage options [10]. As of 2019, California was home to the largest immigrant population in the U.S., including 2.3 million undocumented immigrants [11] most of whom are either Latinx or Asian individuals [12]. California has adopted several immigrant-inclusive policies to promote healthcare access overall and SRH. Examples include the expansion of SRH services via the Family Planning, Access, Care, and Treatment (Family PACT) program, which provides comprehensive family planning access and care regardless of legal status [13]. Additionally, in 2020, eligibility for Medicaid in California, also known as Medi-Cal, was expanded to include low-income, undocumented adults between the ages of 19 to 26, including those with Deferred Action for Childhood Arrivals (DACA) status [14]. Since 2024, all low-income undocumented Californians were eligible for Medi-Cal through the full rollout of Medi-Cal Adult Expansion [15]. Although this expansion is set to be rolled back in 2026 due to budget deficits, California, has historically had a more inclusive health care environment for undocumented immigrants, but there is very little empirical data on this population to understand the effects on sexual and reproductive healthcare use.

Most extant literature examining SRH inequities among immigrants identify differences across nativity (U.S.-born vs. foreign-born) [4]. National datasets such as the National Survey of Family Growth (NSFG) collects data on SRH of immigrants and racial/ethnic minorities and find that immigrants, compared to US-born counterparts, are less likely to use SRH services (69% vs. 73%), have no health insurance coverage (33% vs. 16%), and more likely to have to pay out of pocket for SRH (28% vs. 16%). A systematic review of publicly-funded services providing sexual and reproductive healthcare found that immigrant women with access to Medicaid/Children’s Health Insurance Program (CHIP) had higher rates of prenatal care compared to those without insurance; most studies reviewed focus on Latinx populations [16]. Existing national data, however, do not include legal status and therefore little is known about SRH among the undocumented population.

The studies find that expansion of comprehensive sexual and reproductive healthcare coverage improves access for undocumented immigrants. For example, studies found that a policy expanding access to contraception for all low-income individuals regardless of citizenship status resulted in improved access to moderately and highly effective contraceptive methods [17] and post abortion contraception [18]. Other studies found that long-term effects of expanded Medicaid pregnancy coverage to undocumented immigrants in California improved birth outcomes [19], while state public insurance improved prenatal care utilization [20]. However, there have been notable exclusions in the literature. First, it is important to note that within the undocumented population, researchers also need to consider those with Deferred Action for Childhood Arrivals (DACA). This is an Executive Order signed in 2012 to defer deportation and provide a work permit to about 1.7 million undocumented young people in the US [21]. Small studies have found that 80% of those with undocumented status reported that their legal status affects how they access SRH, while DACA recipients were more likely to report using contraception every time compared to non-recipients [22]. However, the study referenced did not provide comprehensive data on SRH use, payment information, or place of sexual and reproductive healthcare use, which is critical to informing intervention strategies. Second, most studies have focused on Latinx populations. It is also important to include Asian immigrants, given that they are the second largest undocumented group in the US [23]. Studies that use comprehensive SRH measures among the undocumented population are needed.

The objective of this study is to examine the SRH outcomes of undocumented immigrants in California, and in particular the role of health insurance on SRH use. In particular, we hypothesized that undocumented immigrants who were uninsured would have lower sexual and reproductive healthcare use compared to those with private insurance.

## Methods

This study uses data from the community-engaged mixed-methods study, Bridging community, Raising All immigrant Voices for Health Equity (BRAVE).

### Participants

Participants of the study were recruited through 55 community partners (e.g. schools, community-based organizations, and advocacy groups) using email listservs, flyers, and social media. The final sample included 18–40 year old, undocumented female or gender non-conforming immigrants who were living in California at the time of the study and participated in both the baseline and follow-up survey. The current study only uses data from the one-year follow-up survey because SRH questions were only asked in the follow-up survey (*N* = 123).

### Data Collection

Data collection occurred online from July 2020 to February 2021 at baseline and February 2022 to May 2022 in a 1-year follow-up. The study survey was password-protected to minimize fraudulent responses. Participants received a $20 gift card for their participation at follow-up.

### Measures

Measures included history with SRH utilization. “Ever sought SRH advice from a primary care doctor or other health professional” was assessed as a dichotomous measure. We used measures of sexual and reproductive health reflecting comprehensive preventive SRH used in national surveys [4, 24]. In particular, respondents indicated whether they received a pap smear, pelvic exam, STI test, contraception, pregnancy test, or an abortion in the past 12 months (0 = no, 1 = yes). Each of these measures were combined into a cumulative measure of receiving any SRH service from a healthcare provider in the past 12 months. “Ever use a modern method of contraception” included individuals who had used any modern method of contraception^1^ including barrier methods (condom and sponge), short acting reversible methods (SARCs such as pills or ring), long-acting reversible methods (such as IUD or implant), emergency contraception, and/or permanent methods, regardless of past sexual history.

Use of a modern method of contraception at last sex was assessed using a multiple response measure that included a list of several method(s). This measure was recoded into a five-level categorical variable based on the longest-acting method reported by the respondent (0 = none, 1 = natural method (responses included withdrawal or calendar method), 2 = barrier method (condom or sponge), 3 = SARC (pill or ring), 4 = LARC (IUD or implant), and 5 = permanent method (e.g. male partner vasectomy)). The variable was recoded as a binary measure for the multivariate analysis (0 = none and natural methods, 1 = barrier method, SARC, LARC, and permanent methods).

Respondents indicated if they had received the contraception method that they used at last sex. This measure was recoded into a 3-item categorical measure which included private clinics (e.g. employer or company clinic, HMO facility, in-store health clinic, or private doctor’s office), public clinics (e.g. community health clinic, family planning or Planned Parenthood, school, or school-based clinic), and other (e.g. partner or spouse, mail or internet order). “Used insurance to pay for contraception used at last sex” was assessed using an item in which respondents indicated how they paid for their contraception (0 = did not use insurance (or no payment required or some other way), 1 = used insurance). Use of insurance to pay for the contraception used at last sex was assessed as a binary measure (1 = used insurance (e.g. Medicaid or co-payment), 0 = used some other method of payment or no payment was required).

Our main predictor was insurance status. This was examined as a three-category measure of public health insurance (e.g. Medicaid, MediCal, UC SHIP, and Family Health Insurance Plan; and Private as reference category), private health insurance (private or employer-provided insurance), and uninsured (no insurance). DACA status was assessed using a dichotomous measure (0 = no DACA; 1 = DACA (modal category). Missing responses were recoded into the modal category except for DACA status. Respondents missing on DACA status (*N* = 2) were recoded as non-DACA recipients as undocumented immigrants who are not on DACA may be more vulnerable and reluctant to disclose their status [25]. Several sociodemographic factors, such as age, race/ethnicity (Asian and/or Latinx), educational attainment, and employment status were also accounted for in the analysis. Asian respondents indicated ethnicities that included Chinese, Indonesian, Filipino, Indian, Korean, Japanese, and Taiwanese; Latinx respondents indicated countries of birth that included Argentina, Brazil, Colombia, El Salvador, Guatemala, Honduras, Mexico, Panama, Paraguay, Peru, and Venezuela.

### Analysis

Descriptive and multivariate regression analysis were conducted for this study. First, sociodemographic characteristics of the sample and their SRH utilization and outcomes are described. Second, adjusted multivariate logistic regressions were used to assess the role of health insurance on various SRH utilization outcomes in this sample.

All participants provided consent and study materials were reviewed and approved by the Institutional Review Board at the University of California, Los Angeles.

## Results

Sociodemographic characteristics for the total sample are presented in Table 1.

### Sexual and Reproductive Healthcare Use and Outcomes

Approximately 71.5% had ever had sex. Of these, 63% had sex in the past 12 months, 9% had a prior pregnancy, and of those who had a pregnancy, 82% reported that their pregnancy was mistimed and only 3% of the sample had one or more children (Table 2). Nearly half reported that they did not plan to get pregnant. Additionally, 37% reported that their pregnancy plans were influenced by their immigration status.

Half (49%) had ever sought SRH advice from their primary care doctor or other health professional, and among those who did, 85% felt that the advice that they had received was adequate. Approximately 55% had received any SRH services in the past 12 months (i.e. 33% pap smear, 23% pelvic exam, 34% STI test, 34% contraception, 15% pregnancy test, and 2% abortion). Reasons for not receiving any SRH services included not feeling comfortable (44%), cost of care (25%), not having a primary care doctor (28%), having no insurance (21%), lack of time (32%), and not needing or wanting care or because one was not sexually active (14%).

Approximately 94% had ever used a modern method of contraception. The most common method used at last sex was barrier method (40%), followed by none (23%), SARC (17%), LARC (14%), and permanent contraception (1%). At last sex, 35% obtained their method from a public clinic and 32% from a private clinic. Approximately 40% used health insurance to purchase contraception.

### Multivariable Associations with Sexual and Reproductive Healthcare Use and Outcomes

In multivariable results, compared to being privately insured, being uninsured was associated with lower odds of receiving SRH advice from a primary care provider or other healthcare provider (aOR: 0.070; 95% CI: 0.012–0.389) (Table 3; Model 1); having public health insurance and being uninsured significantly reduced the likelihood of receiving any SRH in the past 12 months (aOR_public insurance_: 0.182; 95% CI: 0.053–0.623; aOR_uninsured_: 0.055; 95% CI: 0.012–0.256). Moreover, compared to those with private insurance, those who were uninsured were more likely to obtain contraception from a private clinic, although this was only marginally significant (aOR: 0.108; 95% CI: 0.008–1.384). Compared to those with private insurance, having public health insurance and being uninsured also was associated with lower odds of using health insurance to pay for contraception used at last sex (aOR_public health insurance_: 0.197; 95% CI: 0.043–0.912; aOR_uninsured_: 0.133; *p* < 0.10, 95% CI: 0.015–1.160) (Table 3; Model 4).

DACA status nearly tripled the odds of receiving SRH advice from a primary care or other healthcare provider compared to those without DACA (aOR: 2.775, *p* < 0.10, 95% CI: 0.927–8.308) though the association indicated marginal significance (*p* < 0.10). No other covariates were significant for receiving SRH advice. Compared to completing high school, having a college degree significantly increased the odds of receiving SRH in the past 12 months (aOR: 6.033; 95% CI: 1.716–21.22) (Table 3; Model 2). No other covariates were significant for receiving SRH. Asian respondents had lower odds of obtaining contraception from a private clinic compared to Latinx respondents, although this was only marginally significant (aOR: 0.099, *p* < 0.10, 95% CI 0.010–1.005) (Table 3; Model 3). No other covariates were significant for place of obtaining contraception. Asian respondents had lower odds of using health insurance to pay for contraception used at last sex compared to Latinx respondents (aOR: 0.135; 95% CI: 0.022–0.830).

## Discussion

This study provides comprehensive sexual and reproductive healthcare data among undocumented immigrants. We found that 55% of undocumented participants used SRH in the past 12 months. Past research has found that undocumented immigrants experience poorer access to healthcare and outcomes compared to their documented peers [5, 26–28]. Our study revealed that participants did not receive SRH services due to not feeling comfortable, cost of care, not having a primary care doctor, having no insurance, and lack of time. Unequal access to SRH is associated with adverse birth outcomes [29], maternal mortality [30], serious medical and psychiatric complications [31], and economic insecurity and hardship [32].

This study found that health insurance status was consistently associated with SRH outcomes among those who are undocumented. Compared to having private insurance, being uninsured was associated with lower likelihood of ever seeking SRH advice from a health provider and receiving any SRH in the past 12 months. These findings suggest that insurance status is critical to SRH outcomes. Particularly for younger undocumented immigrants, access to student healthcare via higher education institutions provide considerable efforts in improving SRH access among undocumented immigrants in California [33]. Other studies found that higher education improved knowledge of insurance coverage requirements for preventive and contraceptive services [34–37]. Our study highlights the importance of continuing to expand health insurance at the state and federal level. Our study found that those with public insurance (e.g. Medi-Cal) were also less likely to receive any SRH or use insurance to pay for contraception compared to those with private insurance. This may be counterintuitive in California, where Medi-Cal provides extensive SRH coverage. Studies have found that Medicaid expansions under the Affordable Care Act (ACA) have been associated with greater access and use of highly effective methods of contraception [38, 39] and programs such as California’s Family PACT program [13] have worked to close gaps in SRH for uninsured individuals [40]. This suggests that in addition to policies that promote health insurance and coverage, other factors also need to be addressed within the undocumented community such as mistrust of healthcare institutions [41] and low perceived quality of SRH providers [42].

The DACA program, in providing access to employment healthcare benefits as well as in-state tuition in California to support access to higher education and thus student health plans, has reduced barriers to healthcare for undocumented immigrants [43], and has also been associated with a reduced likelihood of teenage pregnancy [44] and unprotected sex [44], and increased consistent contraception use [22]. In line with this research, our study found that DACA recipients were more likely to seek SRH advice from a health provider compared to those without DACA; however, these findings were not significant. Future studies may wish to explore the intersections of DACA and insurance status on SRH.

The study provides further insight into disparities that Asian women immigrants face in accessing and utilizing SRH, expanding on the current known information about other minorities. The findings provide preliminary evidence that Asian immigrants are less likely than Latinx immigrants to obtain contraception from a private clinic or use insurance to pay for contraception. Past studies on Asian undocumented immigrants have found unique stressors to Asian undocumented communities such as high stigma associated with undocumented status and mistrust in communities, which may result in lack of access to sexual and reproductive healthcare [45]. Data on DACA applications also reveal that Asian immigrants are less likely to apply despite being eligible compared to other ethnicities [46, 47]. Future studies should continue to examine how race/ethnicity intersect with immigration status to shape SRH service and contraception use.

The current study has a number of limitations. First, the outcome data in this study is cross-sectional, meaning no causal claims can be made. Future studies should focus on longitudinal data or collection of life history measures across the life course to establish more robust temporal relationships between insurance status, legal status, and healthcare use over time. Additionally, the study sample was a small convenience sample that was recruited through community partners. Therefore, participants of this study may be better-connected to health services and resources compared to most other undocumented immigrants and lead to conservative estimates for SRH inequities due to recruitment from school and community-based sampling. Future studies may wish to use non-convenience sampling methodologies, such as starfish sampling, which leverages the strengths of time location sampling (TLS) and respondent-driven sampling (RDS) for surveying hidden populations [48]. This sampling approach may be particularly salient for invisibilized populations, or those who are under-represented or excluded from data, such as undocumented populations or those with language-specific barriers [49]. We also were not able to distinguish whether SRH was obtained in the US or their country of origin. Additionally, we combined all Asian ethnicities and Latinx ethnicities into categories, but these groups have incredible heterogeneity across languages, immigration histories, and socioeconomic statuses. Moreover, we reported on marginally significant findings (*p* < 0.10) due to limited sample size and that results are exploratory findings; however, it should be noted that marginally significant findings should be interpreted with caution and to not overinterpret these associations. Related, there was a small sample of those who experienced previous pregnancies and therefore sample sizes were too small to conduct robust statistical analyses on indicators such as pregnancy intentions. Future studies should recruit sufficient samples and provide disaggregated data across racial and ethnic groups to examine intra-group differences, including those with language-specific barriers, in addition to recruiting sufficient samples of birthing people to examine various reproductive health outcomes such as pregnancy intentions. Finally, given that California provides a more inclusive state policy environment for undocumented immigrants compared to most other states, our results are not generalizable to other state contexts.

Despite the limitations, this study offers a number of important insights. First, SRH measures within this study are comprehensive SRH measures validated from national surveys [24]. Additionally, our study includes direct measures of legal and documentation status, unlike national surveys that are known for using algorithms to approximate the undocumented population [44]. We also include a small sample of Asian immigrants to examine heterogenous experiences of undocumented groups. Future studies should focus on more robust sampling methods with a larger sample size and diverse state policy contexts. This includes samples powered to assess different ethnicities within Asian and Latinx populations.

Centering the lived experiences of undocumented immigrants is important for ensuring sexual and reproductive health equity. This study suggests that health insurance plays a critical role in seeking SRH advice, receiving SRH, and having financial support to pay for services. Policymakers should promote policies that expand health coverage to undocumented immigrants to ensure they receive healthcare that is needed.

## Figures and Tables

**Table 1:** Sociodemographic characteristics of undocumented adults ages of 18–40 in California from the BRAVE Study, 2022 (*N* = 123)

	*N*	(%)
Age		
18–24	83	(67.5)
25–31	38	(30.9)
32+	2	( 16)
Gender		
Woman	118	(96.7)
Other*	4	( 3.3)
Race		
Latinx	100	(81.3)
Asian	23	(18.7)
Enrolled in school	78	(63.4)
Educational attainment		
Completed high school or equivalent	26	(21.1)
Some college/community college	22	(17.9)
Four-year college or university	63	(51.2)
Graduate or professional school	12	(9.8)
Employed	85	(69.1)
DACA recipient	82	(66.7)
Speaks English at home	109	(88.6)
Type of health insurance		
None	18	(14.6)
Public	64	(52.0)
Private	41	(33.3)

Notes:

* includes gender non-binary or non-conforming

**Table 2:** Sexual and reproductive health and healthcare outcomes of undocumented adults ages of 18–40 in California from the BRAVE Study, 2022 (*N* = 123)

	*N*	(%)
***Sexual and reproductive health outcomes***		
Ever had sex	88	(71.5)
Had sex in the past 12 months	77	(62.6)
Had ever been pregnant	11	( 8.9)
Pregnancy was mistimed (*N* = 11)	9	(81.8)
Has children	4	(3.3)
***Pregnancy intentions***		
Future pregnancy plans		
I do not plan on getting pregnant at any time in the future	56	(45.5)
I would like to get pregnant in the next 5–10 years, but not before then	39	(31.7)
I would like to get pregnant within 2–5 years, but not in the next year	28	(22.8)
Immigration status affects pregnancy intentions	46	(37.4)
***Sexual and reproductive healthcare use***		
Ever sought SRH advice from primary care doctor or other health professional	60	(48.8)
Received adequate SRH advice (*N* = 60)	51	(85.0)
SRH utilization in the past 12 months	68	(55.3)
Pap smear	40	(32.5)
Pelvic exam	28	(22.8)
STI test	42	(34.1)
Contraception	42	(34.1)
Pregnancy test	18	(14.6)
Abortion	3	( 2.4)
Reasons for not seeking care (*N* = 57)		
Do not feel comfortable	25	(43.8)
Cost of care	14	(24.5)
No primary care doctor	16	(28.1)
No insurance	12	(21.1)
No time	18	(31.6)
Do not need or want care or not sexually active	8	(14.0)
***Contraception outcomes***		
Have you ever used any modern method of contraception (*N* = 88)	83	(94.3)
Type of contraception method used at last sex (*N* = 88)		
Permanent contraception	1	( 1.1)
Long-acting reversible contraception	12	(13.6)
Short-acting reversible contraception	15	(17.0)
Barrier method	35	(39.7)
Natural methods	5	( 5.7)
None	20	(22.7)
Location contraception used at last intercourse was obtained (*N* = 63)		
Community health clinic, community clinic, public health clinic	5	( 7.9)
Drug store	11	(17.5)
Employer or company clinic	1	( 1.6)
Family planning or Planned Parenthood clinic	8	(12.7)
HMO facility	1	( 1.6)
In-store health clinic (like CVS, Target, or Walmart)	9	(14.3)
Mail order/internet	3	( 4.8)
Partner or spouse	4	( 6.4)
Private doctor’s office	9	(14.3)
School or school-based clinic	9	(14.3)
Other	3	( 4.8)
Location contraception used at last intercourse was obtained (*N* = 63)^a^		
Public clinic	22	(34.9)
Private clinic	20	(31.8)
Other	21	(33.3)
Used insurance to pay for contraception used at last intercourse (*N* = 63)	25	(39.7)

a. Public clinics include community health clinics, community clinics or public health clinics, family planning or Planned Parenthood Clinics and school or school-based clinics; private clinics include employer or company clinics, HMO facilities, in-store health clinics, and private doctor’s offices; other includes drug stores, mail or internet order, partner or spouse, or unspecified

**Table 3:** Multivariate binomial logistic regression analysis of correlates of sexual and reproductive healthcare outcomes among undocumented adults (ages of 18–40) in California from the BRAVE Study

	Model 1		Model 2		Model 3		Model 4	
	Ever sought SRH advice from a primary care provider or other healthcare professional		Received any SRH in the past 12 months		Place contraception used at last sex as obtained (private clinic vs. public clinic and other)		Used insurance to pay for contraception used at last sex	
	*N* = 123		*N* = 123		*N* = 63		*N* = 63	
	OR	95% CI	OR	95% CI	OR	95% CI	OR	95% CI
Type of insurance^b^								
Public	0.756	(0.253–2.262)	**0.182** **	(0.053–0.623)	0.398	(0.087–1.815)	**0.197** *	(0.043–0.912)
Uninsured	**0.070** **	(0.012–0.389)	**0.055** ***	(0.012–0.256)	0.108†	(0.008–1.384)	0.133†	(0.015–1.160)
Ages 25–40 (ref. 18–24)	1.001	(0.348–2.881)	0.485	(0.153–1.533)	0.467	(0.103–2.107)	1.005	(0.255–3.969)
Asian (ref. Latinx)	0.680	(0.209–2.212)	0.600	(0.157–2.296)	0.099†	(0.010–1.005)	**0.135** *	(0.022–0.830)
Educational attainment (ref. completed high school)								
Some college^a^	2.247	(0.596–8.474)	2.044	(0.509–8.220)	0.421	(0.050–3.542)	0.323	(0.035–2.980)
College or more	2.671	(0.821–8.690)	**6.033** **	(1.716–21.220)	0.709	(0.121–4.166)	0.706	(0.122–4.081)
Employed	1.694	(0.562–5.109)	1.627	(0.516–5.129)	0.531	(0.125–2.260)		
DACA recipient	2.775†	(0.927–8.308)	2.250	(0.755–6.710)	1.435	(0.297–6.923)	1.489	(0.367–6.038)

(a) includes community college;

(b) health insurance, reference is private health insurance;

*** *p* < 0.001,

** *p* < 0.01,

* *p* < 0.05,

† *p* < 0.10;

Due to low sample size of unemployed individuals, “employment status” was dropped as a predictor for Model 4

## Data Availability

The data is publicly unavailable to protect study participants and confidentiality.

## Footnotes

1 Contraception pills; Condom; Partner’s vasectomy; Female sterilizing operation, such as tubal; Sterilization; Withdrawal, pulling out; Depo-Provera, injectables (shots); Hormonal implants (Norplant or Implanon, or Nexplanon); Calendar rhythm, Standard Days, or Cycle Beads method; Safe period by temperature or cervical mucus test (Two Day, Billings Ovulation, or Sympto-termal method); Diaphragm; Female condom, vaginal pouch; Foam, Jelly or cream; Cervical cream; Suppository, insert; Today sponge; IUD; Lunelle injectable (monthly shot); Contraceptive patch (Ortho-Evra or Xulane); Vaginal contraceptive ring (Nuva RIng); Other method; No other methods ever used.

## References

[1] Hasstedt K, Desai S, Ansari-Thomas Z. Immigrant Women’s Access to Sexual and Reproductive Health Coverage and Care in the United States. Issue Brief Commonw Fund. 2018;2018:1–10. 30458586

[2] Livingston G. Immigrant moms boosted births in 48 states since 1990. Pew Res Cent. 2017. https://www.pewresearch.org/fact-tank/2017/08/29/over-the-past-25-years-immigrant-moms-bolstered-births-in-48-states/ (accessed July 28, 2021).

[3] Barriers to sexual. and reproductive health services faced by immigrant women of reproductive age in the United States. Ibis Reproductive Health; 2023.

[4] Tapales A, Douglas-Hall A, Whitehead H. The sexual and reproductive health of foreign-born women in the United States. Contraception. 2018;98:47–51. 10.1016/j.contraception.2018.02.003. 29453946 PMC6029875

[5] Desai S, Samari G. COVID-19 and Immigrants’ Access to Sexual and Reproductive Health Services in the United States. Perspect Sex Reprod Health. 2020;52:69–73. 10.1363/psrh.12150. 32530526 PMC7307065

[6] Novak NL, Geronimus AT, Martinez-Cardoso AM. Change in birth outcomes among infants born to Latina mothers after a major immigration raid. Int J Epidemiol. 2017;46:839–49. 10.1093/ije/dyw346. 28115577 PMC5837605

[7] Toomey RB, Umaña-Taylor AJ, Williams DR, Harvey-Mendoza E, Jahromi LB, Updegraff KA. Impact of Arizona’s SB 1070 immigration law on utilization of health care and public assistance among Mexican-origin adolescent mothers and their mother figures. Am J Public Health. 2014;104(Suppl 1):S28–34. 10.2105/AJPH.2013.301655. 24354823 PMC3924594

[8] Rhodes SD, Mann L, Simán FM, Song E, Alonzo J, Downs M, The Impact of Local Immigration Enforcement Policies on the Health of Immigrant Hispanics/Latinos in the United States. Am J Public Health. 2014;105:329–37. 10.2105/AJPH.2014.302218. PMC4318326 25521886

[9] Fox A, Howell FM, Weber E, Janevic T. Left Behind: Medicaid Immigrant Exclusions and Access to Maternal Health Care Across the Reproductive-Perinatal Continuum. Med Care Res Rev. 2023;80:582–95. 10.1177/10775587231170066. 37191341

[10] De Trinidad Young M-E, Wallace SP. Included, but Deportable: A New Public Health Approach to Policies That Criminalize and Integrate Immigrants. Am J Public Health. 2019;109:1171–6. 10.2105/AJPH.2019.305171. 31318585 PMC6687277

[11] Perez CA, Mejia MC, Hans J. Immigrants in California. Public Policy Inst Calif; 2021.

[12] Nayak SS, Cardone A, Soberano K, Dhond M. The Health Status of Undocumented Immigrants from Asian Countries in the United States: A Scoping Review and Recommendations for Future Directions. J Immigr Minor Health. 2024;26:1099–112. 10.1007/s10903-024-01625-2. 39180638 PMC11607055

[13] Early DR, Dove M, Thiel de Bocanegra H, Schwarz EB. Publicly Funded Family Planning: Lessons From California, Before And After The ACA’s Medicaid Expansion. Health Aff (Millwood). 2018;37:1475–83. 10.1377/hlthaff.2018.0412. 30179554

[14] Brief PPICP. accessed November 7: Pandemic Changes to Medi-Cal and Implications for California’s Immigrant Farmworkers. Public Policy Inst Calif n.d. https://www.ppic.org/publication/policy-brief-pandemic-changes-to-medi-cal-and-implications-for-californias-immigrant-farmworkers/ (2023).

[15] Dietz M, Lucia L, Kadiyala S, Challenor T, Rak A, Chen Y, California’s Uninsured in 2024: Medi-Cal expands to all low-income adults, but half a million undocumented Californians lack affordable coverage options. Berkeley, CA: UC Berkeley Labor Center; 2023.

[16] Jain T, LaHote J, Samari G, Garbers S. Publicly-Funded Services Providing Sexual, Reproductive, and Maternal Healthcare to Immigrant Women in the United States: A Systematic Review. J Immigr Minor Health. 2022;24:759–78. 10.1007/s10903-021-01289-2. 34697702 PMC10373793

[17] Cohen MA, Boniface ER, Skye M, Linz R, Pedhiwala N, Rodriguez MI. Association of State Funding for Comprehensive Reproductive Health Care With Use of Contraception Among Latina Patients and Non-Latina Patients in Oregon 2023. 10.1001/jamahealthforum.2023.2144 PMC10383011 37505490

[18] Rodriguez MI, Skye M, Shokat M, Linz R, Pedhiwala N, Darney BG. Expanded Access to Postabortion Contraception under Oregon’s Reproductive Health Equity Act. Womens Health Issues. 2022;32:20–5. 10.1016/j.whi.2021.10.001. 34753627

[19] Miller S, Wherry L, Aldana G. Covering Undocumented Immigrants: The Effects of a Large-Scale Prenatal Care Intervention 2022. 10.3386/w30299

[20] Wherry LR, Fabi R, Schickedanz A, Saloner B. State And Federal Coverage For Pregnant Immigrants: Prenatal Care Increased, No Change Detected For Infant Health. Health Aff (Millwood). 2017;36:607–15. 10.1377/hlthaff.2016.1198. 28373325

[21] MPI. Deferred Action for Childhood Arrivals (DACA) Data Tools. MigrationpolicyOrg 2020. https://www.migrationpolicy.org/programs/data-hub/deferred-action-childhood-arrivals-daca-profiles (accessed July 28, 2020).

[22] Sudhinaraset M, Choi HY, Nakphong MK, Woofter R, Brindis CD. Contraceptive use and consistency and the role of deferred action for childhood arrivals: A cross-sectional survey of undocumented young adults. Sex Reprod Healthc. 2022;32:100725. 10.1016/j.srhc.2022.100725. 35533466 PMC12208176

[23] Passel JS, Krogstad JM, in the U.S. Pew Res Cent. What we know about unauthorized immigrants living 2024. https://www.pewresearch.org/short-reads/2024/07/22/what-we-know-about-unauthorized-immigrants-living-in-the-us/ (accessed September 6, 2024).

[24] National Survey of Family Growth. NSFG - Questionnaires, Datasets, and Related Documentation 2020. https://www.cdc.gov/nchs/nsfg/nsfg_questionnaires.htm (accessed September 20, 2021).

[25] Sanchez LE. Exclusion by design: The undocumented 1.5 generation in the U.S. Front Sociol. 2023;8:1082177. 10.3389/fsoc.2023.1082177. 36960305 PMC10029723

[26] Jones J. Current Contraceptive Use in the United States, 2006–2010, and Changes in Patterns of Use Since 1995 2012:26. 24988814

[27] Kim TY, Dagher RK, Chen J. Racial/Ethnic Differences in Unintended Pregnancy: Evidence From a National Sample of U.S. Women. Am J Prev Med. 2016;50:427–35. 10.1016/j.amepre.2015.09.027. 26616306

[28] Institute of Medicine, Committee on the Consequences of Uninsurance. Who Goes Without Health Insurance? Who Is Most Likely to Be. Uninsured? National Academies Press (US); 2001.

[29] Sudhinaraset M, Vilda D, Gipson JD, Bornstein M, Wallace ME. Women’s Reproductive Rights Policies and Adverse Birth Outcomes: A State-Level Analysis to Assess the Role of Race and Nativity Status. Am J Prev Med. 2020;59:787–95. 10.1016/j.amepre.2020.07.025. 33067070 PMC7683369

[30] Ahmed S, Li Q, Liu L, Tsui AO. Maternal deaths averted by contraceptive use: an analysis of 172 countries. Lancet Lond Engl. 2012;380:111–25. 10.1016/S0140-6736(12)60478-4. 22784531

[31] Ogbu-Nwobodo L, Shim RS, Vinson SY, Fitelson EM, Biggs MA, McLemore MR, Mental Health Implications of Abortion Restrictions for Historically Marginalized Populations. N Engl J Med. 2022. 10.1056/NEJMms2211124. 36300980

[32] Foster DG, Biggs MA, Ralph L, Gerdts C, Roberts S, Glymour MM. Socioeconomic Outcomes of Women Who Receive and Women Who Are Denied Wanted Abortions in the United States. Am J Public Health. 2022;112:1290–6. 10.2105/AJPH.2017.304247. 35969820 PMC9382171

[33] ACHA. 2019 Sexual Health Services Survey Survey Report. American College Health Association; 2020.

[34] Long M, Frederiksen B, Ranji U, Diep K, Salganicoff A. Many Women Use Preventive Services, but Gaps in Awareness of Insurance Coverage Requirements Persist: Findings from the 2022 KFF Women’s Health Survey. Kaiser Family Foundation; 2023.

[35] Sutton MY, Anachebe N, Lee R, Skanes H. Racial and Ethnic Disparities in Reproductive Health Services and Outcomes, 2020. Obstet Gynecol. 2021;137:225–33. 10.1097/AOG.0000000000004224. 33416284 PMC7813444

[36] Becker NV, Polsky D. Women Saw Large Decrease In Out-Of-Pocket Spending For Contraceptives After ACA Mandate Removed Cost Sharing. Health Aff (Millwood). 2015;34:1204–11. 10.1377/hlthaff.2015.0127. 26153316

[37] Guttentag S, The Annual Cost of Birth Control. GoodRx Health. 2021. https://www.goodrx.com/conditions/birth-control/annual-cost-of-birth-control (accessed May 12, 2023).

[38] Ranji U, Long M, Salganicoff A, Silow-Carroll S, Rosenzweig C, Rodin D, Beyond the Numbers: Access to Reproductive Health Care for Low-Income Women in Five Communities. San Francisco, CA: Kaiser Family Foundation; 2019.

[39] Darney BG, Jacob LR, Hoopes M, Rodriguez MI, Hatch B, Marino M, Evaluation of Medicaid expansion under the Affordable Care Act and contraceptive care in US community health centers. JAMA Netw Open. 2020;3:e206874. 10.1001/jamanetworkopen.2020.6874. 32496568 PMC7273194

[40] Women’s Preventive Services Guidelines. Hum Resour Serv Adm 2022. https://www.hrsa.gov/womens-guidelines-2016 (accessed May 12, 2023).

[41] Sudhinaraset M, Ling I, Gao L, Chavarin J, Gee GC. The association between Deferred Action for Childhood Arrivals, health access, and mental health: the role of discrimination, medical mistrust, and stigma. Ethn Health 2020:1–13. 10.1080/13557858.2020.1850647 33276705

[42] Sudhinaraset M, Woofter R, Huang P. Stratified reproduction and threats to reproductive justice for immigrants: Decision-making for fertility using an ecological model. Soc Sci Med. 2025;382:118334. 10.1016/j.socscimed.2025.118334. 40577942 PMC13170025

[43] Woofter R, Sudhinaraset M. Differences in Barriers to Healthcare and Discrimination in Healthcare Settings Among Undocumented Immigrants by Deferred Action for Childhood Arrivals (DACA) Status. J Immigr Minor Health. 2022. 10.1007/s10903-022-01346-4. PMC9256563 35226220

[44] Kuka E, Shenhav N, Shih K. A Reason to Wait: The Effect of Legal Status on Teen Pregnancy. AEA Pap Proc. 2019;109:213–7. 10.1257/pandp.20191013.

[45] Sudhinaraset M, Ling I, To TM, Melo J, Quach T. Dreams deferred: Contextualizing the health and psychosocial needs of undocumented Asian and Pacific Islander young adults in Northern California. Soc Sci Med 1982. 2017;184:144–52. 10.1016/j.socscimed.2017.05.024. 28527372

[46] Singer A, Svajilenka N. Immigration Facts: Deferred Action for Childhood Arrivals (DACA). Washington DC: Brookings; 2013.

[47] Wong TK, García AS, Does Where I, Live Affect Whether I. Apply? The Contextual Determinants of Applying for Deferred Action for Childhood Arrivals. Int Migr Rev. 2016;50:699–727. 10.1111/imre.12166.

[48] Raymond HF, Chen Y-H, McFarland W. Starfish Sampling: a Novel, Hybrid Approach to Recruiting Hidden Populations. J Urban Health. 2019;96:55–62. 10.1007/s11524-018-0316-9. 30328063 PMC6391284

[49] Sudhinaraset M, Kim H, Song K, Ronquillo RJ, Kim J, Raymond HF. Implementation of starfish sampling for invisibilised populations: a methods protocol of the BRAVE multi-site cross-sectional community-based participatory study. BMJ Open. 2025;15:e108572. 10.1136/bmjopen-2025-108572 PMC12716530 41419291

